# Known-group validity of passive knee joint position sense: a comparison between individuals with unilateral anterior cruciate ligament reconstruction and healthy controls

**DOI:** 10.1186/s13018-023-03996-y

**Published:** 2023-07-22

**Authors:** Mustafa Jebreen, Nicola Maffulli, Filippo Migliorini, Ashokan Arumugam

**Affiliations:** 1 Department of Physiotherapy, College of Health Sciences, University of Sharjah, Sharjah, United Arab Emirates; 2 Physiotherapy and Rehabilitation Department, Sheikh Shakhbout Medical City, Abu Dhabi, United Arab Emirates; 3 Department of Medicine, Surgery and Dentistry, University of Salerno, Baronissi, SA Italy; 4 Faculty of Medicine, School of Pharmacy and Bioengineering, Keele University, Stoke on Trent, England; 5 Barts and the London School of Medicine and Dentistry, Centre for Sports and Exercise Medicine, Mile End Hospital, Queen Mary University of London, London, England; 6 Department of Orthopaedic, Trauma, and Reconstructive Surgery, RWTH University Hospital of Aachen, 52074 Aachen, Germany; 7 Neuromusculoskeletal Rehabilitation Research Group, RIMHS–Research Institute of Medical and Health Sciences, University of Sharjah, Sharjah, United Arab Emirates; 8 Sustainable Engineering Asset Management Research Group, RISE-Research Institute of Science and Engineering, University of Sharjah, Sharjah, United Arab Emirates; 9 Department of Physiotherapy, Adjunct Faculty, Manipal College of Health Professions, Manipal Academy of Higher Education, Manipal, Karnataka India; 10 Department of Orthopaedics and Trauma Surgery, Academic Hospital of Bolzano (SABES-ASDAA), 39100 Bolzano, Italy

**Keywords:** Joint position sense, Anterior cruciate ligament, Psychometric properties, Discriminative validity, Known-groups validity

## Abstract

**Background:**

Knee joint position sense (JPS) might be negatively affected after injuries to the anterior cruciate ligament (ACL). Recent systematic reviews suggest further investigation of psychometric properties, including validity, of knee JPS tests following ACL reconstruction (ACLR). This study investigated the known-group validity by comparing knee JPS errors between individuals who underwent unilateral ACLR and healthy controls.

**Methods:**

This cross-sectional study involved 36 men, including 19 after ACLR (ACLR group) and 17 healthy controls (control group). In both groups, the absolute error (AE), constant error (CE) and variable error (VE) of passive knee JPS were calculated in the flexion and extension directions, for two target angles (30° and 60° flexion) per direction. Discriminative validity was evaluated by comparing JPS errors between the operated and non-operated knees in the ACLR group. Known-group validity was evaluated by comparing JPS errors between the operated knees in the ACLR group and the asymptomatic non-dominant knees of healthy controls.

**Results:**

Mean AE, CE and VE for all tests were 4.1°, − 2.3° and 3.6° for the operated knees in the ACLR group, 5.5°, − 2.6° and 3.3° for the non-operated knees in the ACLR group and 4.6°, − 2.6° and 3.3° for the non-dominant knees in the control group, respectively, regardless of the test direction and target angle. The operated knees in the ACLR group did not show significantly greater JPS errors compared to the contralateral knees in the ACLR group and to the non-dominant knees in the control group (*p* ≥ 0.05). On the other hand, the non-operated knees showed significantly greater AE for the 0°–60° flexion test (*p* = 0.025) and CE for the 0°–30° flexion test (*p* = 0.024) than the operated knees in the ACLR group. JPS errors did not significantly differ in the operated knees in the ACLR group based on the direction of movement and the target angle. However, the errors were significantly higher when the knee was moved through a greater range compared to that of a lesser range between the starting and target angles.

**Conclusion:**

The ACLR knees did not show greater passive JPS errors than the contralateral or control knees. The direction of movement and target angle did not influence the JPS acuity after ACLR. However, higher JPS errors were evident when the knee was moved through a greater range compared to a lesser range of motion. Further studies investigating the psychometric properties of standardized JPS tests following ACLR are warranted.

**Supplementary Information:**

The online version contains supplementary material available at 10.1186/s13018-023-03996-y.

## Background

Anterior cruciate ligament (ACL) injuries are common, accounting for 68.6 isolated injuries per 100,000 person-years in the USA [1]. Most ACL injuries are non-contact and occur during dynamic knee valgus motion [2, 3]. Proprioceptive deficits are considered an intrinsic predisposing factor to ACL injuries [4]. Treatment strategies include rehabilitation with or without ACL reconstruction (ACLR) [5]. However, despite treatment, approximately 1 in 4 young athletes who sustain an ACL injury and return to high-risk sports sustain a secondary ACL injury [6].

Mechanoreceptors have been identified in the ACL [7–10], suggesting an important proprioceptive function for this ligament in the knee joint [11]. Injury to the ACL affects the mechanoreceptors which provide proprioceptive information about the knee joint. Therefore, joint stability, biomechanics and neuromuscular control will be adversely affected [12–15], leading to an increased risk of re-injury and delayed return to play [16]. Addressing proprioception during post-surgical rehabilitation and/or ACL injury prevention programs may improve proprioceptive acuity and neuromuscular control, restore stability and decrease the risk of re-injury, and might aid successful return to play [12, 14, 15, 17, 18].

Assessing knee proprioception after an ACL injury is clinically important to guide the rehabilitation program [19–21]. Joint position sense (JPS) is a part of proprioception, and it consists of the individual’s ability to recognize joint position in space [19–22]. JPS is assessed by measuring the acuity of reproducing a defined target angle (angle reproduction) [20, 21, 23], actively or passively. Active reproduction relies mainly on the afferent information from the muscle spindles and allows to compensate the proprioceptive loss in the affected knee. The passive method relies more on the afferent output from the proprioceptors located inside that knee and therefore more precisely assesses JPS acuity [22, 24]. JPS error can be reported in different ways: absolute error (AE), constant error (CE) and variable error (VE) [25]. AE is the most commonly reported error in the literature. It represents the absolute amount of deviation of each reproduced angle from a given target angle regardless of the direction of deviation, and provides an estimate of performance accuracy. The CE represents the amount and direction of deviation of each reproduced angle from a given target angle, and provides an estimate of performance bias. The VE represents the variability of the reproduced angles about the individual’s average score, irrespective of the proximity to the target angle, and provides an estimate of performance consistency [25]. Agreement on which outcome measures to use while quantifying the JPS error is still lacking. However, other variables, in addition to the method of reproduction (active or passive) and the outcome measure (AE, CE or VE), should be considered when assessing different testing protocols of knee JPS: testing instrument (e.g., electrogoniometer or dynamometer), joint loading (weight bearing or non-weight bearing), testing position (lying, sitting, or standing), reproduction method (ipsilateral or contralateral), the direction of movement (extension or flexion), range of movement (starting angle to target angle) [19, 22].

Sound clinical decision-making depends, among others, on the psychometric properties of applied measurement tools and associated outcomes. Clinicians and researchers need tools with sufficient validity when quantifying knee joint proprioception after ACLR. Unless knee proprioceptive measurements are valid and reliable (with low standard errors of measurement and minimal detectable change scores), assessing clinically meaningful changes in proprioception over time in individuals with and without proprioceptive deficits is difficult. Systematic reviews and meta-analyses [21, 22, 26, 27] have reported a lack of well-defined psychometric properties of proprioceptive assessment tools, including those for individuals after ACLR. A recent meta-analysis found that ACLR knees showed significantly greater JPS error when compared to asymptomatic controls (*p* = 0.002), based on 13 studies with most of them rated with “doubtful” risk of bias [22] when assessed using the consensus-based standards for the selection of health measurement instruments (COSMIN) risk of bias checklist tool [28]. Their subgroup analysis based on the method of reproduction revealed a non-significant difference between groups for passive reproduction (*p* = 0.67). Moreover, the authors [22] highlighted the poor level of reporting the methods and outcomes across the studies which investigated knee JPS after ACL injuries.

Based on the current evidence and suggested recommendations [21, 22, 26, 27], high-quality research is warranted to substantiate the level of evidence of psychometric properties of knee JPS tests for individuals following ACLR. Therefore, the primary aim of this study was to evaluate the AE, CE and VE for passive knee JPS tests in both flexion and extension directions of the operated knees in individuals who underwent an ACLR and compare them to the contralateral non-operated knees of the same individuals (discriminative validity) as well as to the asymptomatic knees of healthy controls (known-groups validity). The secondary aim of the study was to investigate any possible influence of the direction of movement, target angle and range of movement during the test on knee proprioceptive acuity.

## Methods

### Study design

This cross-sectional observational design was conducted at the Physiotherapy and Rehabilitation Department of Healthpoint Hospital in Abu Dhabi, United Arab Emirates. The Ethics approval was obtained from the research ethics committee of Healthpoint Hospital (ID: MF2467-2021-13). All participants signed a written informed consent before data collection.

### Participants sampling and recruitment

A convenience sample of 36 participants, including 19 participants who had undergone ACLR surgery (ACLR group) and 17 healthy controls (control group), completed this study. The ACLR group were active young men who underwent primary unilateral ACLR with a hamstring autograft during the previous 12 months and were undergoing or had completed post-operative rehabilitation following the surgery. ACLR group’s participants were excluded if they were older than 35 years or younger than 18 years, were within the first 3 months of rehabilitation post-surgery, had undergone a concomitant cartilage procedure and/or other ligament reconstruction, had undergone other operations of the lower limbs within a year before the ACLR, had knee pain, swelling and/or fear of movement, indicated by a score of > 37 on the Tampa scale for kinesiophobia (TSK) [29], which prevented completion of the test. Age- and activity-level-matched healthy participants were recruited if they had no knee pain or history of knee injury/surgery.

All participants who had undergone ACLR and followed a standard post-operative protocol at the hospital were recruited for the study. Only patients who underwent at least ACLR 3 months before the study were eligible. This rehabilitation protocol consisted of six phases and started on the second day after surgery and continued until the time to return to normal activity, including sports. Progression from one phase to the next depended on passing specific criteria rather than the time duration after surgery. This supervised program required individuals to attend the rehabilitation department twice weekly, supplemented with an independent home exercise program performed daily by the participants.

Neuromuscular and proprioceptive exercises were an integral part of the protocol, and they were gradually commenced after the first phase of rehabilitation (3 weeks post-surgery) and continued to the end of the rehabilitation program. Closed kinetic chain exercises, among others, began with simple weight shifting between legs in bipedal stance, and unipedal stance, and then progressed to exercises performed on the biomedical ankle platform system (Spectrum Therapy Products, Adrian, MI, USA) and a BOSU ball (BOSU, Ashland, OH, USA). In the more advanced stages of rehabilitation, perturbation training and exercises on a trampoline were performed.

### Instrumentation

Knee JPS was assessed using the Biodex System 4 dynamometer (Biodex Medical Systems, Shirley, NY, USA). Trial-to-trial and day-to-day reliability as well as criterion validity of the Biodex dynamometer for position testing demonstrated excellent values (ICCs 0.99–1.00) [30].

### Procedures

The same physical therapist assessed the participants’ eligibility, calibrated the dynamometer, provided a familiarization session, conducted the tests and collected the data for each participant. Two days before the testing session, each eligible participant was evaluated to ensure his ability to participate in the study. Participants’ demographic and anthropometric data were collected. Additionally, self-reported outcomes (numeric pain rating scale [NPRS] [31], international knee documentation committee [IKDC] [32], TSK [33] and Tegner activity scale [TAS] [34]) were collected to assess the participants’ self-perceived knee symptoms [31–33] and functions [32] and to ensure that both groups have a matched activity level [34]. Next, a familiarization session with a thorough demonstration and protocol explanation was provided.

During the testing session, a random selection for the testing order, to choose the limb, direction of movement and target angle, was conducted by using the “Spin the Wheel—Random Picker” mobile application version 2.5.9 (Taurius Petraitis). The participant wore a blindfold to eliminate any visual input during the test. The participant was seated on the dynamometer chair, with his back supported on an 85° inclined backrest and the popliteal fossa placed approximately 5 cm away from the chair, and the arms crossed on the chest. The thigh of the tested limb was secured to the chair by a strap. The dynamometer lever attachment was secured approximately 5 cm above the lateral malleolus. The dynamometer was calibrated according to the manufacturer's guidelines.

The “extension” test started when the examiner passively moved the participant’s knee from the starting angle (90° flexion) toward a target angle (30° and 60° flexion in random order). The target angle was held for 5 s, and the participant was asked to memorize this angle. The examiner then passively moved the knee to the same starting angle. Thereafter, the dynamometer passively moved the knee toward extension at an angular velocity of 5°/second [35]. The participant was told to press a hold button, placed in his hand, when he perceived that the target angle was reproduced (reproduced angle). Similarly, the “flexion” test followed the same sequence, but the passive movements were from the starting angle (0° flexion) toward a target angle (30° and 60° flexion in random order). Six trials [36] were repeated to reproduce each target angle in each direction, totaling 24 trials per knee. Table 1 shows the parameters of the testing protocol. The testing protocol is provided as a supplementary video file (Additional file 1).

**Table 1:** Joint position test methods

Procedure and outcomes	Remarks	
Method of reproduction	Passive	
Tested limb	Both limbs	
Testing position	Sitting	
Equipment	Biodex dynamometer (system 4)	
Demonstrating side	Ipsilateral	
Direction of knee motion	Extension	Flexion
Starting angle	90°	0°
Target angle	30° and 60°	30° and 60°
Angular velocity	5°/s	
Memorization time for target angle	5 s	
Number of trials per target angle	6	
Outcome measurement	Absolute error Constant error Variable error	

### Outcome measures

The mean AE was calculated using the formula: mean AE = $\frac{{\sum {\text{the}}\left| {{\text{RA}} - {\text{TA}}} \right|}}{n}$, where RA is the reproduced angle, TA is the target angle, and n is the number of trials. The mean CE in flexion direction was calculated using the formula: mean CE (flexion) = $\frac{{\sum {\text{RA}} {-}{\text{ TA}}}}{n}$, while the mean CE in extension direction was calculated using the formula: mean CE (extension) = $\frac{{\sum {\text{TA}} {-} {\text{RA}}}}{n}$. VE was calculated using the formula: VE = $\sqrt { \frac{{\sum \left( {{\text{RA}}i - M} \right)^{2} }}{n}}$, where RA_*i*_ is the score of the *i*th trial of interest and M is the mean reproduced angle of the complete set of trials [25].

### Statistical methods

Data analyses were completed using the IBM SPSS software version 21.0 (IBM Corp., Armonk, NY, USA). Data normality was tested using the Shapiro–Wilk test. If data normality was violated, the Wilcoxon signed ranks and the Mann–Whitney U tests were used instead of paired sample and independent samples t-tests, respectively.

The participants’ baseline characteristics were assessed using the independent samples *t*-test. The paired sample *t*-test was used to assess the discriminative validity by comparing JPS errors between the operated and non-operated knees of the ACLR group. The independent samples *t*-test was used to assess the known-groups validity by comparing JPS errors between the operated knees of the ACLR group and the non-dominant knees of the control group. Additionally, the paired sample *t*-test was used to evaluate the possible effects of the direction of movement, target angle and range of movement during the test on JPS errors for each group. The significance level was set at a *p* value of < 0.05 for all analyses.

## Results

### Participants

No significant differences were found between participants in both groups (*p* ≥ 0.05). Table 2 provides the participants’ characteristics [38].

**Table 2:** Participants’ characteristics

Variable	ACLR group	Control group
Participants, n	19	17
Age (years), median (range)	24 (21–35)	25 (22–35)
BMI (kg/m^2^), mean ± SD	28.3 ± 4.6	26.9 ± 2.8
Time interval between surgery and test (months), mean ± SD	6.3 ± 2.5	-
Self-reported rating scales, median (range)		
TAS (baseline)	7 (6–9)	7 (6–9)
TAS (post)	4 (3–7)	-
NPRS b (rest)	0 (0–1)	-
NPRS (worst)	3 (1–7)	-
IKDC c	72.4 (42.5–94.3)	-
TSK d	25 (20–33)	-

BMI: Body Mass Index; TAS: Tegner Activity Scale; NPRS: Numeric Pain Rating Scale; IKDC: International Knee Documentation Committee; TSK: Tampa Scale for Kinesiophobia

^a^TAS: A self-reported scale (0–10) for assessment of level of knee-demanding activities. A high score indicates an ability to participate in more knee-demanding activities [34]

^b^NPRS: A self-reported scale (0–10) for assessment of pain level. A high score indicates higher level of pain [31]

^c^IKDC: A self-reported scale (0–100) for assessment of the level of symptoms, functions, and sports. A high score indicates higher functions and lower symptoms [32]. An Arabic-translated validated version was used for Arabic-speaking participants [37]

^d^TSK: A self-reported scale (17–68) for assessment of fear of movement. A high score indicates a high fear of movement and re-injury [33]. An Arabic-translated validated version was used

### Discriminative Validity

**Fig. 1:**
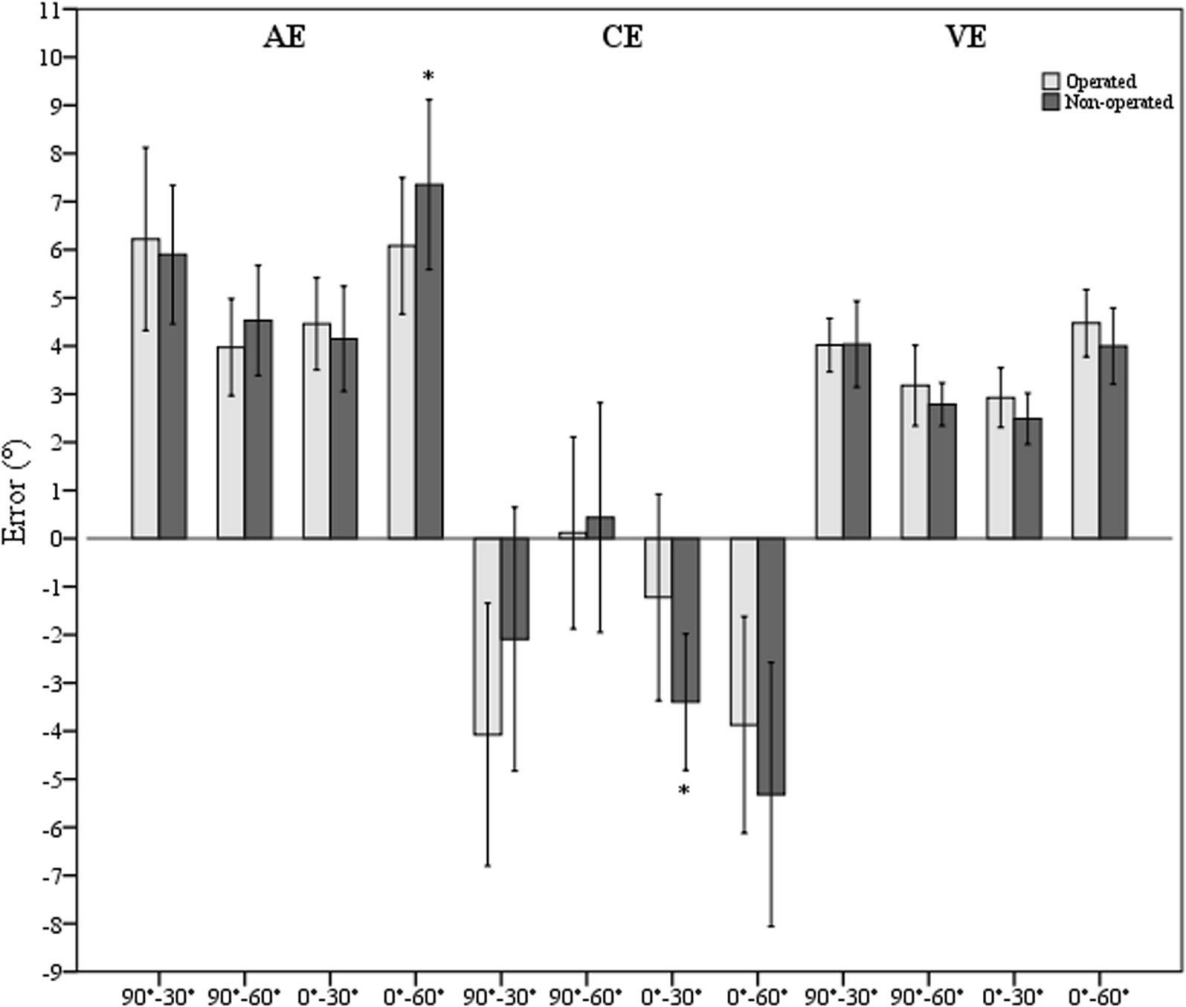
Bar charts with error bars represent mean AE, CE and VE as well as 95% CI for the operated and non-operated knees of the ACLR group. *significant difference (*p* < 0.05)

In the ACLR group, the mean AE, CE and VE for all tests were 4.1°, − 2.3° and 3.6° for the operated knees and 5.5°, − 2.6° and 3.3° for the non-operated knees, respectively, regardless of the test direction and target angle. The operated knees did not show any significantly greater JPS error than the non-operated knees in all tests for all outcome measures. Conversely, the non-operated knees showed significantly greater AE in the 0°–60° test and CE in the 0°–30° test (*p* = 0.025 and 0.024, respectively) (Fig. 1).

### Known-groups validity

**Fig. 2:**
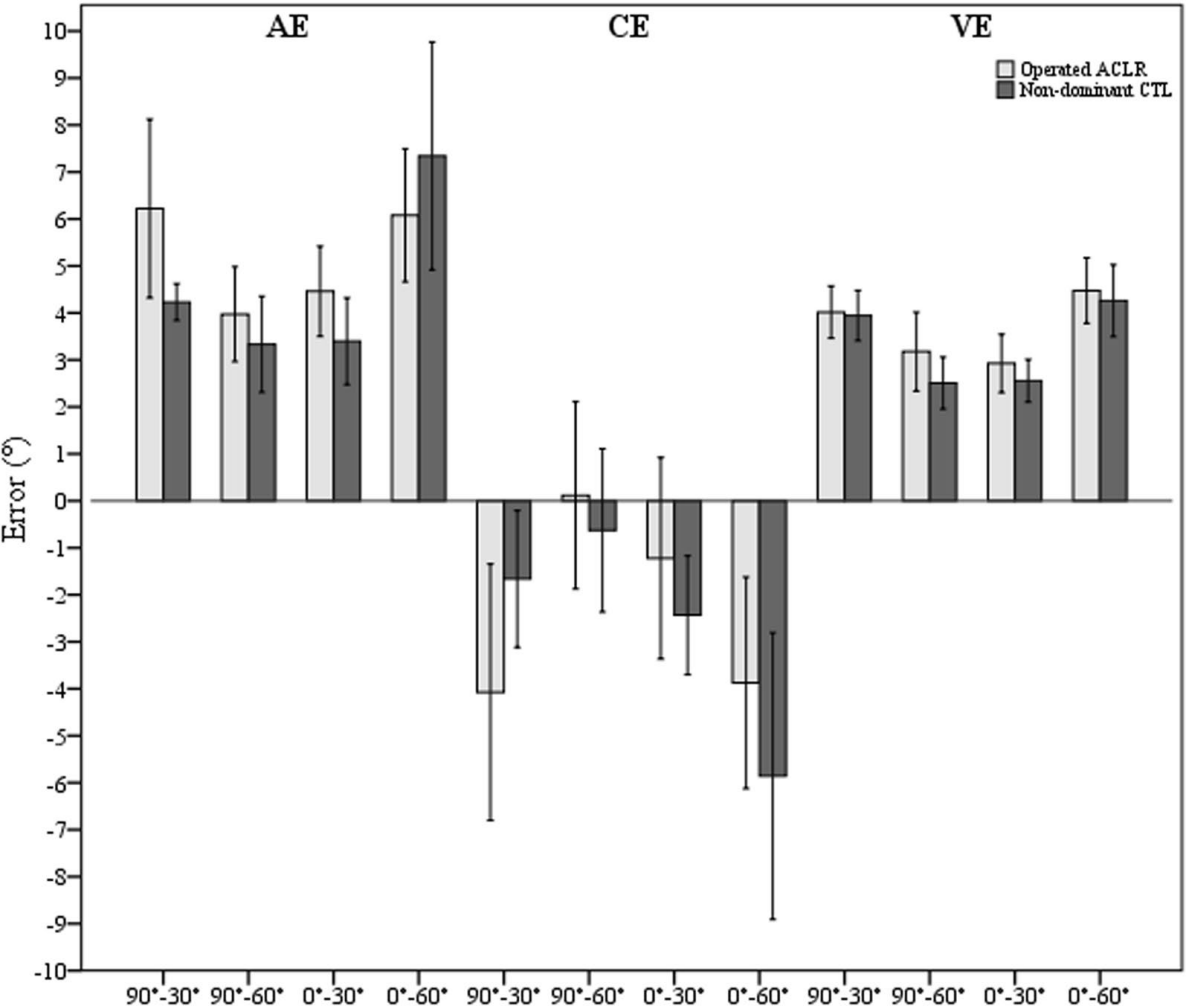
Bar charts with error bars represent mean AE, CE and VE as well as 95% CI for the operated knees of the ACLR group and the non-dominant knees of the control (CTL) group

In the control group, the mean AE, CE and VE for all tests were 5.1°, − 3.0° and 2.6° for the dominant knees and 4.6°, − 2.6° and 3.3° for the non-dominant knees, respectively, regardless the test direction and target angle. There were no JPS differences between the operated knees of the ACLR group and the non-dominant knees of the control group for all outcome measures (Fig. 2).

### Influence of direction, target angle and range of movement during the test

#### Direction of movement (extension vs flexion)

**Fig. 3:**
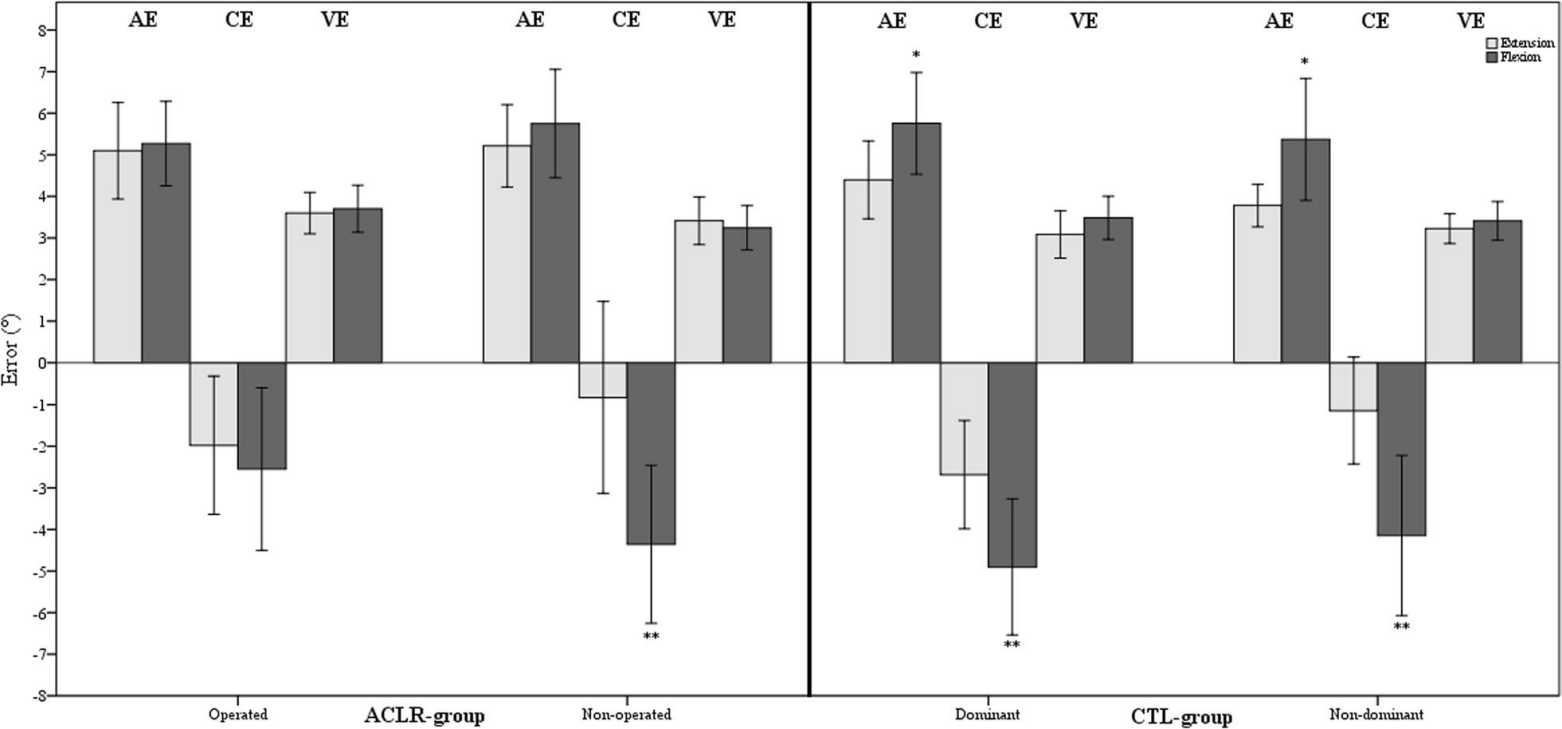
Bar charts with error bars represent the mean AE, CE and VE as well as 95% CI for the extension and flexion tests in both groups. CTL—control. *significant difference (*p* < 0.05). **significant difference (*p* < 0.01)

Extension and flexion directions were studied by comparing the average of the means of both extension tests (90°–30° and 90°–60°) and the average of the means of both flexion tests (0°–30° and 0°–60°) for both groups separately. For the ACLR group, only the non-operated knees showed a greater CE in the flexion direction than the extension direction (− 4.4° ± 3.9°, − 0.8° ± 4.8°; *p* = 0.001). For the control group, both knees showed a greater AE and CE in the flexion direction than the extension direction ([AE: 5.8° ± 2.4°, 4.4° ± 1.8°; *p* = 0.013 and 5.4° ± 2.9°, 3.8° ± 1°; *p* = 0.035], [CE: − 4.9° ± 3.2°, − 2.7° ± 2.5°; *p* = 0.001 and − 4.1° ± 3.7°, − 1.1° ± 2.5°; *p* = 0.002], for the dominant and non-dominant knees, respectively) (Fig. 3).

#### Target angle (30° vs 60°)

**Fig. 4:**
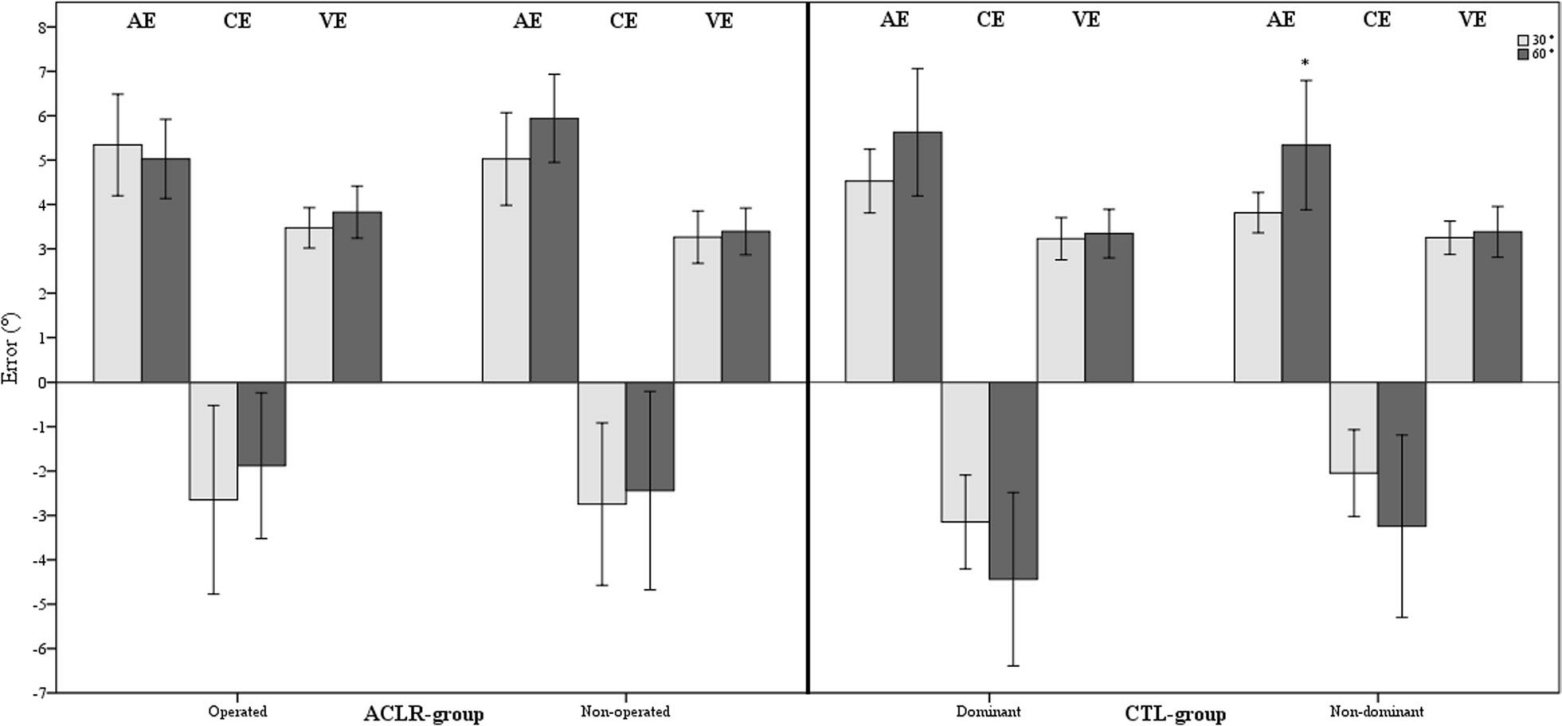
Bar charts with error bars represent the mean AE, CE and VE as well as 95% CI for the tests with 60° and 30° target angles in both groups. CTL—control. *significant difference (*p* < 0.05)

Both target angles were analyzed by comparing the average of means of both tests whose target was 30° (90°–30° and 0°–30°) to the average of means of both tests whose target was 60° (90°–60° and 0°–60°). Both knees of the participants who underwent ACLR did not show significant differences in JPS based on the target angle. In the control group, the non-dominant knees showed a greater AE during the test with 60° target angle than the tests with 30° target angle (5.3° ± 2.8°, 3.8° ± 0.9°; *p* = 0.032, for both target angles, respectively) (Fig. 4).

#### Range of motion during the test (60° vs 30°)

The ranges moved during the test were investigated by comparing the average of the means of both tests whose angular displacement was 60° (90°–30° and 0°–60°) to the average of the means of both tests whose angular displacement was 30° (90°–60° and 0°–30°). The mean AE, CE and VE were significantly higher when the knee was moved through a greater range (60°) compared to when it was moved through a lesser range (30°), for both groups and both sides. Table 3 illustrates the level of significance for these comparisons.

**Table 3:** Mean error for tests with 60° and 30° angular displacement

Range moved during the test	ACLR group					
	Operated knee			Non-operated knee		
	AE	CE	VE	AE	CE	VE
60°	6.2° ± 2.8°	− 4.0° ± 4.3°	4.2° ± 0.9°	6.6° ± 2.5°	− 3.7° ± 4.9°	4.0° ± 1.4°
30°	4.2° ± 1.6°	− 0.5° ± 3.6°	3.1° ± 1.3°	4.3° ± 1.9°	− 1.5° ± 3.4°	2.6° ± 0.9°
*P* value	0.005	0.002	0.005	0.001	0.007	< 0.001

	Control group					
	Dominant knee			Non-dominant knee		
	AE	CE	VE	AE	CE	VE
60°	6.4° ± 2.9°	− 5.2° ± 3.3°	4.1° ± 1.5°	5.8° ± 2.3°	− 3.8° ± 3.7°	4.1° ± 0.9°
30°	3.7° ± 1.4°	− 2.3° ± 2.8°	2.5° ± 0.7°	3.4° ± 1.5°	− 1.5° ± 2.5°	2.5° ± 0.8°
*P* value	0.001	0.001	< 0.001	< 0.001	0.013	< 0.001

*AE* absolute error, *CE* constant error, *VE* variable error

## Discussion

This study primarily aimed to evaluate the mean AE, CE and VE for passive knee JPS tests of the operated knees in individuals after a unilateral ACLR and compare the results to the contralateral knees (discriminative validity) and to age- and activity level-matched controls (known-group validity). Effects of movement direction, target angle and range of movement during the test on knee JPS acuity were secondarily investigated. Participants scored an average AE of 4.1°, 5.5° and 4.6° for the operated, contralateral and control knees, respectively. These scores are in accordance with the average ranges identified by a recent meta-analysis [22] for similar individuals based on the studies that used the passive JPS tests after an ACL injury; the ACLR knees scored an average of 0.8°–10.18°, the contralateral knees scored an average of 0.63°–6.74° and the knees of the healthy controls scored an average of 0.98°–9.65°. The knee which had undergone ACLR did not show significantly greater JPS errors compared to the contralateral knees and to healthy controls.

The non-significant differences between the operated knees and the contralateral or healthy knees can be explained by understanding that part of the proprioceptive loss after an ACL injury results from the altered kinematics of the joint. Therefore, reconstructing the ACL may successfully restore its mechanical function and improve knee proprioception [39, 40]. The regeneration process of sensory neurons in the graft, proved by the detectable somatosensory evoked potentials after direct electrical stimulation of the reconstructed ACL [41], may help to enhance the recovery of the proprioceptive function within the knee joint [40, 41]. Additionally, it is possible that the lost proprioceptive signals as a result of the ACL injury can be compensated by the integrated afferent signals arising from the mechanoreceptors located in the other ligaments, joint capsule, surrounding muscles, tendons and skin, as only 1–2.5% [8, 9] of the total ACL area is composed of mechanoreceptors [27]. Also, training and physical activity may induce some peripheral and central neural adaptations leading to improved proprioception [42]. Therefore, the post-operative exercises, including proprioception exercises, provided to our participants during the rehabilitation program may have influenced their JPS acuity. However, a recent systematic review on the psychometric properties of knee JPS tests [22] concluded that the responsiveness of JPS tests to different interventions was not significantly different in individuals with ACL injury/reconstruction. A lack of statistically significant differences could be due to low sensitivity of different JPS test methods and associated outcome measures (AE, CE or VE) or heterogeneity of interventions reported in the literature. Another systematic review [43] evidenced a low certainty of evidence to substantiate the effects of neuromuscular rehabilitation training on improving knee JPS following ACLR. The authors recommended that novel well-designed neuromuscular training interventions and valid proprioceptive measures are warranted to ascertain definitive evidence in this area. Therefore, we included participants with a unilateral ACLR with a hamstring autograft in the past 12 months irrespective of their stage of rehabilitation.

The presence of JPS deficits after ACLR has been widely debated. Our study is not the only one to have not identified JPS differences between the ACLR knees and the contralateral knees [44–46] or between the ACLR knees and healthy controls [46, 47] when the target angles were passively reproduced and an isokinetic dynamometer was used to evaluate proprioception. Faggal et al. [45] investigated the effects of post-operative proprioceptive training on functional performance, dynamic balance and JPS in individuals who had undergone ACLR with or without stump preservation. They compared the JPS AE between both groups (*n* = 15 per group), 3 months post-reconstruction. The operated and non-operated knees showed non-significant differences for the group with stump preservation (2.38° ± 1.13° and 2.17° ± 1.12°; *p* = 0.245), as well as for the group without stump preservation (2.26° ± 1.99° and 1.66° ± 0.83°; *p* = 0.336). Ordahan et al. [46] tested the JPS in a group of participants with ACLR (*n* = 20), comparing them to a group of healthy controls (*n* = 16), assessing the effectiveness of the post-operative exercises, including proprioception exercises, on knee pain, function and proprioception. There were no AE differences (*p* ≥ 0.05) between ACLR group compared to the contralateral knees and to the healthy controls 6 months after surgery. Littmann et al. [47] studied the presence of proprioceptive impairments in women with ACLR (*n* = 11), and compared them with healthy controls (*n* = 20). The AE of passive JPS did not differ between groups (10.18° ± 6.85° for ACLR group, and 9.65° ± 6.96° for the control group; *p* = 0.21).

Some authors did report statistically significant differences based on similar comparisons. Lee et al. [48] found a significant AE difference between the operated and non-operated sides in 28 individuals whose ACL was reconstructed more than 3 months after injury (7.84° ± 4.22° and 6.74° ± 5.77°; *p* = 0.025), although the comparison revealed a non-significant difference in another 48 individuals whose ACL was reconstructed within 3 months after injury (5.58° ± 4.31° and 5.60° ± 4.95°; *p* = 0.915). Zhou et al. [49] evaluated the JPS 6 months after ACLR to describe the factors influencing proprioception after surgery, and to investigate the association between proprioception and muscle strength. They observed a significant proprioception deficit when comparing the ACLR group (*n* = 36) to healthy controls (*n* = 13). The mean AE was 5.59° ± 2.57° for the ACLR group and 4.34° ± 1.08° in the control group (*p* = 0.023). Variations of the testing methods and variables investigated within the studies of the knee JPS following ACLR can explain the differences of the results [22, 50] between these studies. Testing instrument, method of reproduction, body position, direction and range of movement, angular velocity, target memorization duration, number of trials and reported outcome measures, as well as the participants characteristics including surgical procedure and time elapsed from injury to surgery and from surgery to test, vary widely between the studies.

In the present study, direction of movement and target angle did not influence the JPS acuity in participants with ACLR, although participants in the control group scored greater AE and CE in flexion than extension. These results are in accordance with the results of other studies despite the differences in testing procedures. Mir et al. [51] evaluated the knee JPS during a functional standing weight bearing in individuals with ACLR (*n* = 12) and healthy controls (*n* = 12). They found no significant AE differences based on the direction of movement within or between groups (*p* ≥ 0.05). Similarly, Hopper et al. [52] examined a group of individuals after ACLR (*n* = 9) in a functional standing weight bearing position; the mean AE did not differ between flexion and extension (*p* = 0.47). However, another study [42] investigated JPS in healthy participants to provide normative data from participants aged 18–82 years and evaluate the effects of age, physical activity and motion on knee JPS. Using the active reproduction method, the study found a greater AE in flexion than extension (3.6° ± 1.6° and 2.9° ± 1.5°; *p* = 0.0001). These results are similar to the results of our control group who showed significantly greater AE in flexion than extension for the dominant (5.8° ± 2.4° and 4.4° ± 1.8°; *p* = 0.013) and the non-dominant side (5.4° ± 2.9° and 3.8° ± 1°; *p* = 0.035). Additionally, in our study, the ACLR group and control group showed significantly higher JPS error when the knee was moved through a greater range (60°) compared to when it was moved through a lesser range (30°). Other authors [42, 53, 54] reported similar findings when they investigated the possible effects of the angular displacement during the proprioceptive task on JPS error for different joints in the body of healthy individuals. The reduced JPS acuity associated with the greater range of movement during the test can be explained by the greater cognitive demands needed, which makes the proprioceptive process more complicated thus leading to greater JPS errors [42, 53, 54].

Assessing proprioception after ACLR remains a challenge. Knee proprioception is commonly assessed by the angle reproduction to quantify the JPS, and threshold to detect passive movement (TTDPM) to quantify the joint movement sense, where the person needs only to indicate the first instance that he/she perceives a joint movement [19–22, 50]. TTDPM was found to be a more reliable and valid method that can precisely identify deficits in proprioception following ACLR [40, 50, 55]. Other authors [55] further questioned the clinical relevance of quantifying proprioceptive deficits after ACLR given their low-to-moderate correlation with the knee functional outcomes. They warranted the need to develop relevant tests that are able to evaluate the role of the sensorimotor system after ACLR.

Our study used passive angle reproduction to quantify JPS errors since active reproduction is believed to allow for proprioceptive compensation after ACL injury. Additionally, we evaluated the AE, CE and VE to comprehensively describe the individual’s JPS performance in terms of accuracy (AE), bias (CE) and consistency (VE). Given the conflicting evidence on knee JPS after ACLR, our study compared the results of the ACLR knees to the contralateral knees and healthy controls, as internal and external controls, to better evaluate the proprioceptive functions in these individuals. To maintain consistency, the same therapist conducted the test and assessed all participants. Including female participants was precluded by the cultural barriers in our setting that prevent women from being assessed by a male researcher for a research study; therefore, the results may differ for female participants. We did not perform post-hoc power analysis. Post-hoc power analysis, using the observed effect size, has been criticized [56–62].

## Conclusion

The operated knees of individuals who underwent ACLR did not show greater knee JPS error (AE, CE and VE) than the contralateral non-operated knees or the asymptomatic knees of healthy controls. Direction of movement and target angle did not influence the JPS acuity in the ACLR group. However, both sides of the ACLR group and control group showed higher JPS errors when the knee was moved through a greater range compared to when it was moved through a lesser range. Standardized knee JPS testing protocols, with sufficient level of evidence for their psychometric properties, need to be developed following ACLR.

## Supplementary Information

**Additional file 1**. Passive knee joint position sense testing protocol.

## Data Availability

The data underlying this article are available in the article and in its online supplementary material.

## Abbreviations

ACL: Anterior cruciate ligament
ACLR: Anterior cruciate ligament reconstruction
AE: Absolute error
BMI: Body mass index
CE: Constant error
COSMIN: Consensus-based standards for the selection of health measurement instruments
IKDC: International knee documentation committee
JPS: Joint position sense
NPRS: Numeric pain rating scale
TAS: Tegner activity scale
TSK: Tampa scale for kinesiophobia
TTDPM: Threshold to detect passive movement
VE: Variable error
